# Early changes in transient adenosine during cerebral ischemia and reperfusion injury

**DOI:** 10.1371/journal.pone.0196932

**Published:** 2018-05-25

**Authors:** Mallikarjunarao Ganesana, B. Jill Venton

**Affiliations:** 1 Department of Chemistry, University of Virginia, Charlottesville, VA, United States of America; 2 Department of Chemistry and Neuroscience Graduate Program, University of Virginia, Charlottesville, VA, United States of America; Universita degli Studi di Napoli Federico II, ITALY

## Abstract

Adenosine is an important neuromodulator in the central nervous system, and tissue adenosine levels increase during ischemic events, attenuating excitotoxic neuronal injury. Recently, our lab developed an electrochemical fast-scan cyclic voltammetry (FSCV) method that identified rapid, spontaneous changes in adenosine concentrations that last only about 3 seconds. Here, we investigated the effects of cerebral ischemia and reperfusion on the concentration and frequency of transient adenosine release in the caudate-putamen. In anesthetized rats, data were collected for four hours: two hours of normoxia, 30 min of cerebral ischemia induced by bilateral common carotid artery occlusion, and 90 min of reperfusion. Transient adenosine release was increased during the cerebral ischemia period and remained elevated during reperfusion. The total number of adenosine transients increased by 52% during cerebral ischemia and reperfusion compared to normoxia. The concentration of adenosine per event did not increase but the cumulative adenosine concentration during cerebral ischemia and reperfusion increased by 53% because of the higher frequency of events. Further, we evaluated the role of A_2A_ antagonist, SCH442416, a putative neuroprotective agent to affect adenosine transients. SCH442416 significantly decreased the transient frequency during cerebral ischemia-reperfusion by 27% and the cumulative concentration by 31%. Our results demonstrate that this mode of rapid adenosine release increases during early cerebral ischemia-reperfusion injury. Rapid adenosine release could provide fast, local neuromodulation and neuroprotection during cerebral ischemia.

## Introduction

Ischemic stroke is one of the leading causes of death in major industrialized countries, with a mortality rate of 30%, and results in long term disabilities in survivors [1]. Adenosine is an important inhibitory neuromodulator that increases during stroke and acts as a neuroprotective agent [2–6]. As a breakdown product of ATP, adenosine builds up in the extracellular space during stressful events, [7,8] and reduces excitotoxic glutamate signaling [9]. Much research in this field has focused on which receptors mediate the effects of adenosine and adenosine receptors are important targets for stroke therapies [10–13]. For example, A_1_ agonists potentiate the response of inhibitory A_1_ receptors, while A_2A_ antagonists reduce the immediate excitatory effects of adenosine. In addition to using adenosine receptor agonists and antagonists as treatments for stroke, modulating adenosine itself is a candidate for prophylactic neuroprotection [13]. Adenosine increases during ischemia and stays elevated during reperfusion and this increase has been traditionally measured on the minutes to hours time scale [14–17]. Melani et al. first reported a significant increase in extracellular adenosine levels after *in vivo* ischemia, in awake freely moving rats after permanent middle cerebral artery occlusion [18]. Recent evidence suggests that some adenosine receptors rapidly desensitize and that adenosine is acting on a faster time scale, in the range of seconds not minutes [19–21]. Dale et al. demonstrated continuous increase in extracellular adenosine during a 5 min hypoxic episode in CA1 area of rat hippocampal slices using a biosensor [22]. However, rapid, real-time measurements of adenosine have not been studied during cerebral ischemia and reperfusion.

Our lab has identified a mode of spontaneous, rapid mode of adenosine release that lasts only about three seconds [23–25]. Using fast-scan cyclic voltammetry [26], transient adenosine release spontaneously occurs, with a frequency of every 1–3 minutes depending on brain region. Transient adenosine release is also caused by mechanical stimulation of brain tissue [27]. For example, spontaneous adenosine release has been measured after implantation of deep-brain stimulating electrodes, where it may be protective and contributing to the microthalamotomy “honeymoon” effect of improvement directly after electrode implantation [28,29]. Transient adenosine modulates dopamine neurotransmission but the rapid nature of the neuromodulation demonstrates that transient adenosine release may have different actions than basal changes in adenosine [23–25]. Spontaneous adenosine release during the progression of cerebral ischemia and reperfusion has not been characterized and it could play an important role as a local, rapid neuromodulatory signal.

Here, we examine spontaneous, adenosine transients in real-time during the early stages of cerebral ischemia-reperfusion injury *in vivo*. Cerebral ischemia was induced through transient bilateral common carotid artery occlusion (BCCAO) for 30 minutes, and measurements then taken for 90 minutes of reperfusion. BCCAO is a mild model of transient global hypoperfusion [30] that does not induce a large ischemic insult, due to the presence of efficient collateral systems in the rodent brain, but does casue a detectable change in oxidative stress events [31,32]. Spontaneous adenosine release was measured with FSCV in the caudate-putamen using an implanted carbon-fiber microelectrode. The main finding is that the frequency of adenosine transients increases during cerebral ischemia and reperfusion but the average concentration of each transient remains the same. However, the cumulative concentration of adenosine significantly increased due to the higher frequency of transients. The A_2A_ antagonist SCH442416 significantly decreased the number of transients during cerebral ischemia-reperfusion, showing that this putative neuroprotective drug actually decreased adenosine. This study shows that spontaneous, transient adenosine release is modulated by mild ischemic stress and could provide rapid, local neuromodulation during stroke.

## Materials and methods

### Materials

All reagents were purchased from Sigma Aldrich (St. Louis, MO, USA) unless otherwise noted. Phosphate buffered saline was adjusted to pH 7.4 and was made with sodium chloride (NaCl, 131.25 mM), sodium phosphate (NaH_2_PO_4_, monohydrate, 10.0 mM), potassium chloride (KCl, 3.0 mM) from Fisher Scientific (Fair Lawn, NJ, USA), sodium sulfate (Na_2_SO_4_, anhydrous, 2.0 mM), calcium chloride, (CaCl_2_, dihydrate, 1.2 mM), magnesium chloride (MgCl_2_, hexahydrate, 1.2 mM) (Fisher). DMSO (dimethyl sulfoxide) was purchased from Amresco (Solon, OH, USA). Vascular occluders (VO1.5N) to induce the stroke were obtained from DOCXS biomedical (Ukiah, CA).

The A_2A_ receptor antagonist SCH 442416 (2-(2-Furanyl)-7-[3-(4-methoxyphenyl)propyl]-7*H*-pyrazolo[4,3-*e*][1,2,4]triazolo[1,5-*c*]pyrimidin-5-amine) was purchased from Tocris Biosciences (Ellisville, MO, USA) and was dissolved in 200 μL of DMSO and was administered at 3 mg/kg, i.p.

### Electrodes and fast-scan cyclic voltammetry

Carbon-fiber microelectrodes were prepared as previously described (Huffman & Venton, 2008). In brief, a carbon fiber (T-650, Cytec Engineering Materials, West Patterson, NJ, USA) of 7 μm in diameter was aspirated into a glass capillary (1.2 x 0.68 mm) and pulled using a vertical pipette puller (model PE-21; Narishige, Tokyo, Japan) into two electrodes. The protruding carbon fiber was cut to 100–125 μm with a scalpel. Electrical connection was made by backfilling the capillary with 1 M KCl. The silver-silver chloride reference electrodes were made in house by electrodepositing chloride onto a silver wire (Acros Organics, New Jersey, USA).

Fast-scan cyclic voltammetry (FSCV) was used to detect and quantitate adenosine on a sub-second time scale [26]. The FSCV waveform and data were collected through computer controlled HDCV software (University of North Carolina, Chapel Hill, NC, USA). A Dagan Chem Clamp potentiostat (Dagan Corporation, Minneapolis, MN, USA) was used to apply the potential. The applied waveform was from –0.40 V to 1.45 V and back at 400 V/s, for every 100 milliseconds against Ag/AgCl reference. Applying the waveform produces a large background current, thus data were background subtracted (10 cyclic voltammograms averaged) to remove non-Faradaic currents. Electrodes were post-calibrated for 1.0 μM adenosine in phosphate buffered saline immediately following animal experiments and the currents were used to estimate the concentrations of adenosine *in vivo*.

### Animal experiments and induction of cerebral ischemia

All animal experiments were reviewed and approved by the Institutional Animal Care and Use Committee of the University of Virginia. Animal welfare was monitored daily by animal care staff. Male Sprague-Dawley rats (Charles River Laboratories, Wilmington, MA, USA) between 250–350 grams were housed in 12/12 hour light/dark cycles and fed *ad libitum* and provided environmental enrichment. Surgeries were performed in the morning, during the beginning of the light cycle. Experiments were performed in lab while animals were anesthetized. Animals were assigned to different experimental groups by the experimenter (not randomized), with stroke and control rats interspersed, for experiments. Experiments were performed without any blinding procedure. Study was not pre-registered. Urethane is a commonly used anesthesia for non-survival voltammetry surgery and fits well with our experimental timeline. Prior to the anesthetic injection with urethane (1.5 g/kg, i. p.), rats were initially anesthetized with isoflurane (1 mL/100 g rat weight). Surgical areas were exposed by shaving around the surgical sites.

To facilitate BCCAO, a midline incision was made under the neck to expose the tissue and the right and left common carotid arteries were isolated and exposed. An occluder cuff was placed around each of the exposed arteries and secured in place using a suture material passed through the eyelets of the occluder. The rat was then placed in a stereotaxic frame and 250 μL of bupivacaine (Sensorcaine, MPF, APP Pharmaceuticals, LLC; Schaumburg, IL, USA) was injected subcutaneously at the top of the skull prior to incision. Holes were drilled in the skull for the placement of both working and reference electrodes [33]. The working carbon-fiber microelectrode was placed in the caudate-putamen (in mm from bregma): AP: +1.2, ML: +2.0, DV: –4.5. The Ag/AgCl reference electrode was placed on the contralateral side. The rat’s body temperature was regulated with a temperature controlled heating pad and thermistor probe (FHC; Bowdoin, ME, USA).

A carbon-fiber microelectrode was placed in the caudate-putamen and equilibrated about 30 minutes until a steady background with optimal shape was observed. If fewer than 5 adenosine transients were seen in the first 30 minutes of equilibration (25% of the animals did not meet the criteria), a new electrode was inserted. Data were excluded if the frequency of transient adenosine release was less than ten transients per hour. Once the electrode placement was optimized, data were collected for two hours of normoxia (pre-ischemia) and two hours of cerebral ischemia (30 min) and reperfusion (90 min). cerebral ischemia was induced by inflating the occluders for 30 min, during which the blood flow through both the common carotid arteries was completely blocked. After 30 min of cerebral ischemia, the occluders were deflated, allowing reperfusion and measurements were continued for 90 min. In control group of sham surgery experiments, the same exact procedure was followed as for cerebral ischemia, except the occluders were not inflated. Although the transient frequency/number of transients between rats varies significantly, our protocol facilitates within animal control for all the groups. At the conclusion of experiment, animals were sacrificed through decapitation.

To determine the extent of pathological changes in the neuronal nucleus, brain samples were collected 8 hours after the induction of cerebral ischemia. Rats were perfused with 2.5% glutaraldehyde and 4% formaldehyde in PBS and decapitated to collect the caudate putamen region of the brain samples and were fixed in the same solution for overnight at 4 ^o^C. Samples were washed with PBS after fixing with 1% osmic acid for 1 h. After being embedded in an 100% epon resin, ultra thin sections were cut at 75 nm, picked up on 200 mesh copper grids and stained with 0.25% lead citrate and 2% uranyl acetate. The cortical microvessels were observed under a JEOL 1230 type transmission electron microscope (Electron Co., Japan) equipped with a 4K x 4K CCD camera from SIA and photographed.

For the drug experiments, two hours of pre-drug (normoxia) data were collected, and then the drug was injected right before cerebral ischemia induced and two hours of post-drug data were collected. Principal component regression analysis was used to calculate concentration and frequency of transient adenosine release [23]. A training set was created for each animal using the seven largest transients in the pre-drug data [34]. For some of the data, an automated analysis program was used that identifies random adenosine transients from FSCV data sets [35].

### Statistics

All statistics were performed using GraphPad Prism 6 (GraphPad Software Inc., San Diego, CA, USA). The data were presented as normalized mean ± SEM. A Kolmogorov-Smirnov (KS) test was used to determine underlying distributions between inter-event times (time between consecutive transients). ANOVA was used to compare multiple 30 minute bins, with p-values adjusted down for multiple comparisons. A paired t-test was used to compare the concentration means between two groups. The experiment was exploratory and so predefined effects were not available, however sample sizes were chosen to be able to pick out 30% differences in means. For power analysis, the number of animals per group was calculated based on paired t-test compairson (using MedCalc). For cumulative concentration, the standard deviation of the difference between the first 2 hours and second 2 hours is 2.9 μM. In order to see a minimum difference of 4 μM, with an α = 0.5 and power (1-β) of 0.8, n = 7 samples are needed. All data collected were used and no outliers were identified. All data were considered significant at the 95% confidence level.

## Results

### Detection of adenosine with fast-scan cyclic voltammetry

**Fig 1:**
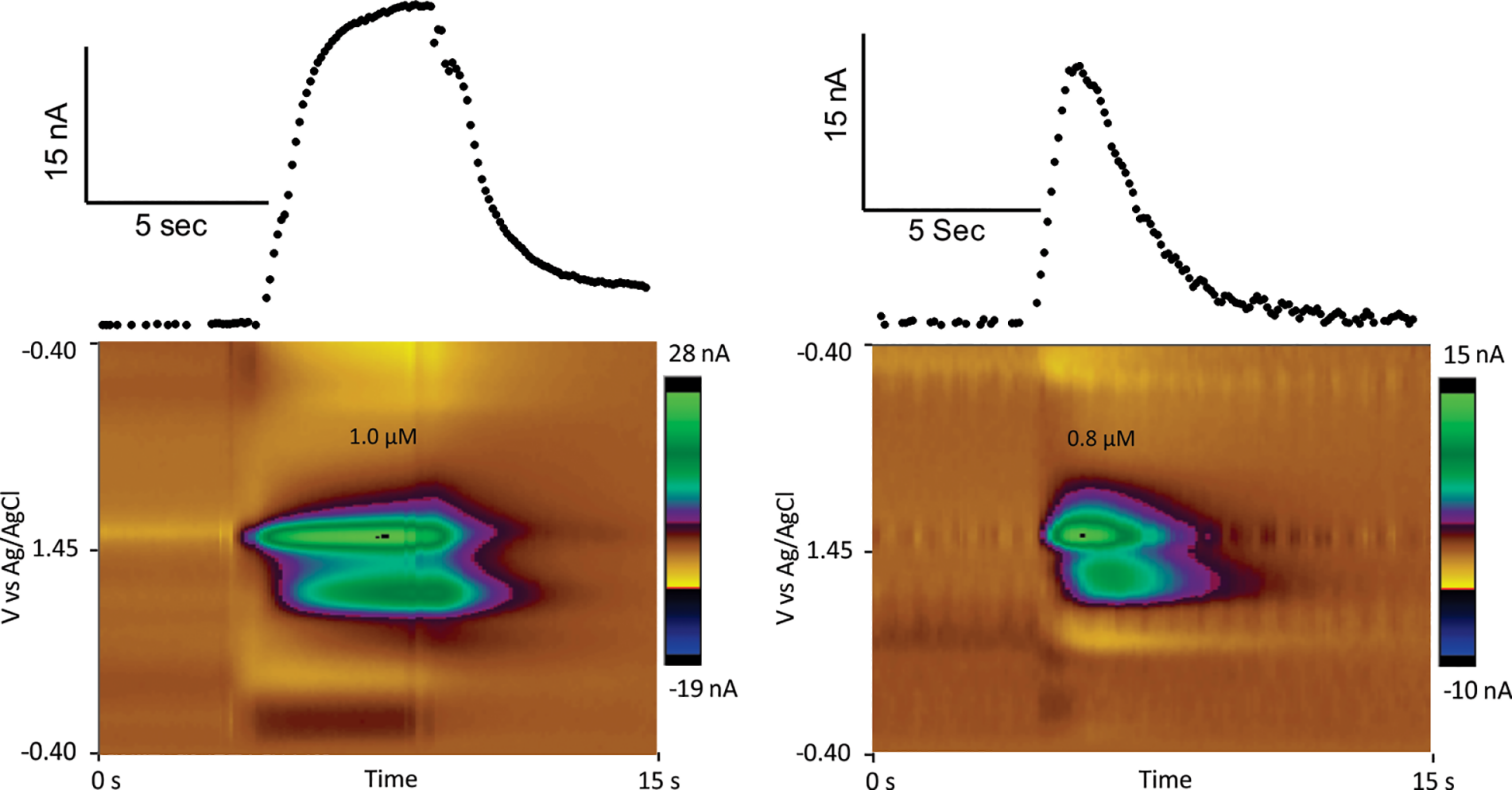
Detection of Adenosine *in vitro* and *in vivo* using fast scan cyclic voltammetry. (A) *In vitro* calibration of adenosine. A 3-D color plot (middle) depicts the time on the x-axis, potential on the y-axis, and current in false color. The primary oxidation at +1.4 V (large green oval in center of color plot) and the secondary oxidation at +1.0 V (green/purple oval below center oval). The current vs time plot (top) shows the change in current in the presence of adenosine. (B) *In vivo* spontaneous, transient adenosine event, detected in rat caudate-putamen. The 3-D color plot shows primary and secondary oxidation peaks that match the in vitro calibration. The current vs time plot (top) shows the change in current due to the spontaneous adenosine transient.

Rapid release of spontaneous, transient adenosine release was measured continuously using FSCV in vivo in anesthetized rats [23]. A carbon-fiber microelectrode was inserted into the caudate-putamen and a waveform was continuously applied. Adenosine is oxidized via two sequential, two-electron oxidation steps that produce both a primary oxidation peak at 1.4 V and a secondary oxidation peak at 1.0 V [26]. FSCV data are commonly plotted in three dimensional color plots to show changes in adenosine oxidation over time. Here, in the example color plots (Fig 1, bottom) the primary oxidation peak is seen as the green/purple circles in the center of the plot, while the circle directly below at 1.0 V is the secondary peak, which slightly delays the primary peak. The top plot shows changes at the primary adenosine oxidation potential, converted to concentration, over time.

**Fig 2:**
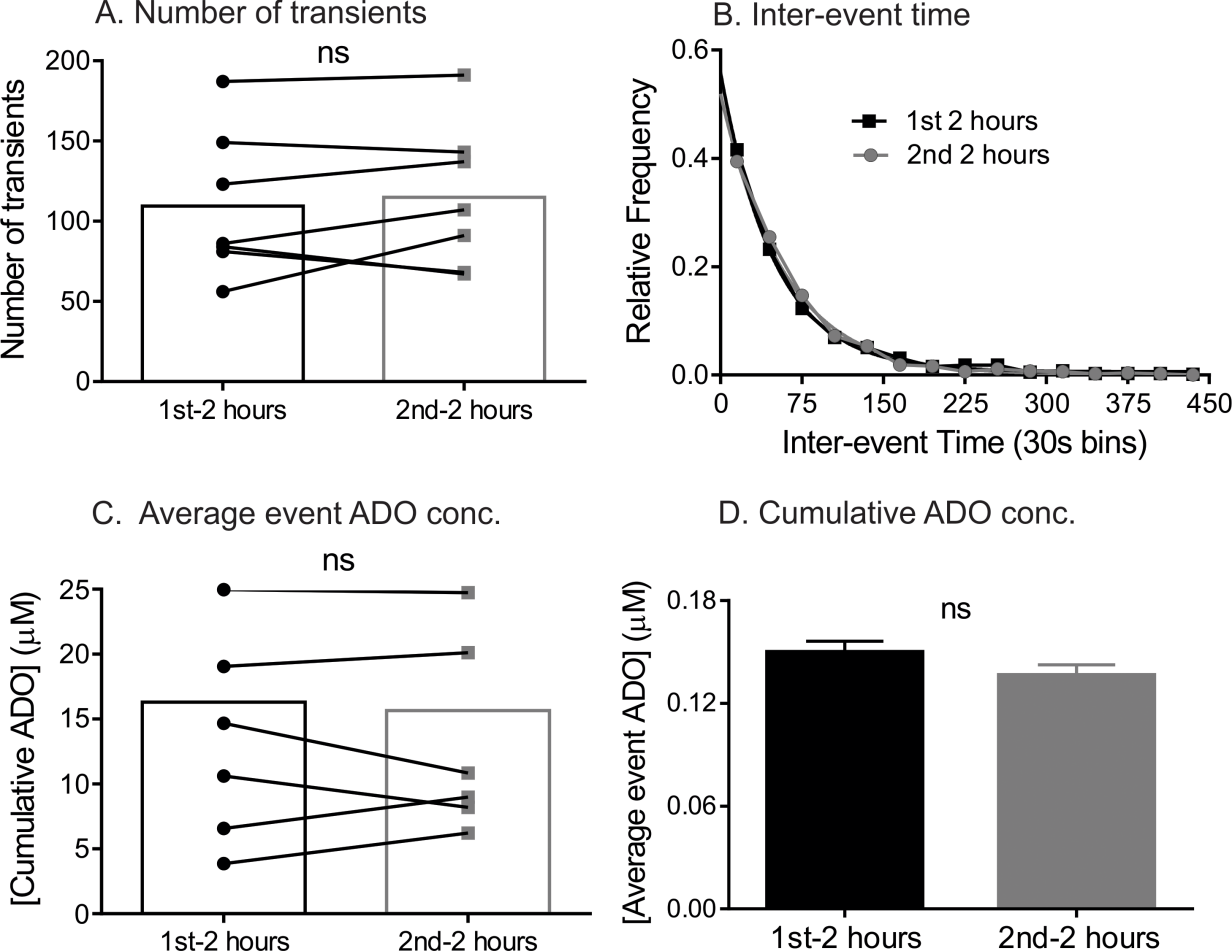
Control experiments with no ischemia. All data and statistics are for n = 7 animals (A) Number of adenosine transients did not change between the 1^st^ and 2^nd^ 2 hour periods (paired t-test, p = 0.48). (B) Inter-event time of all adenosine transients. The exponential fit (black line) in the first 2 h is y = 0.5592e^-0.0207x^ (R^2^ = 0.99) and in the second 2 h (grey line) is y = 0.5195e^-0.0172x^ (R^2^ = 0.99). There was no significant difference between the underlying distributions in the first 2 h and second 2 h (KS-test, n = 7 animals, p = 0.6). (C) The average event adenosine concentration per transient. There was no significant change in the average adenosine concentration per transient for first 2 hrs (n = 1084) and second 2 hrs (n = 1064) (unpaired t-test, n = 7 animals, p = 0.12). (D) There was no significant change in the mean cumulative concentration between the 1^st^ and 2^nd^ 2 hour periods (paired t-test, p = 0.55).

As a control, one group was given sham surgery, and adenosine was continuously monitored for 4 hours, without inflating the occluders. The number of transients in the 1st 2 hours was not significantly different than the 2nd 2 hours, indicating no changes over 4 hours (Fig 2). Also there was no change in inter-event time, average event concentrations, or cumulative adenosine concentrations.

### Real-time monitoring of adenosine during cerebral ischemia-reperfusion

**Fig 3:**
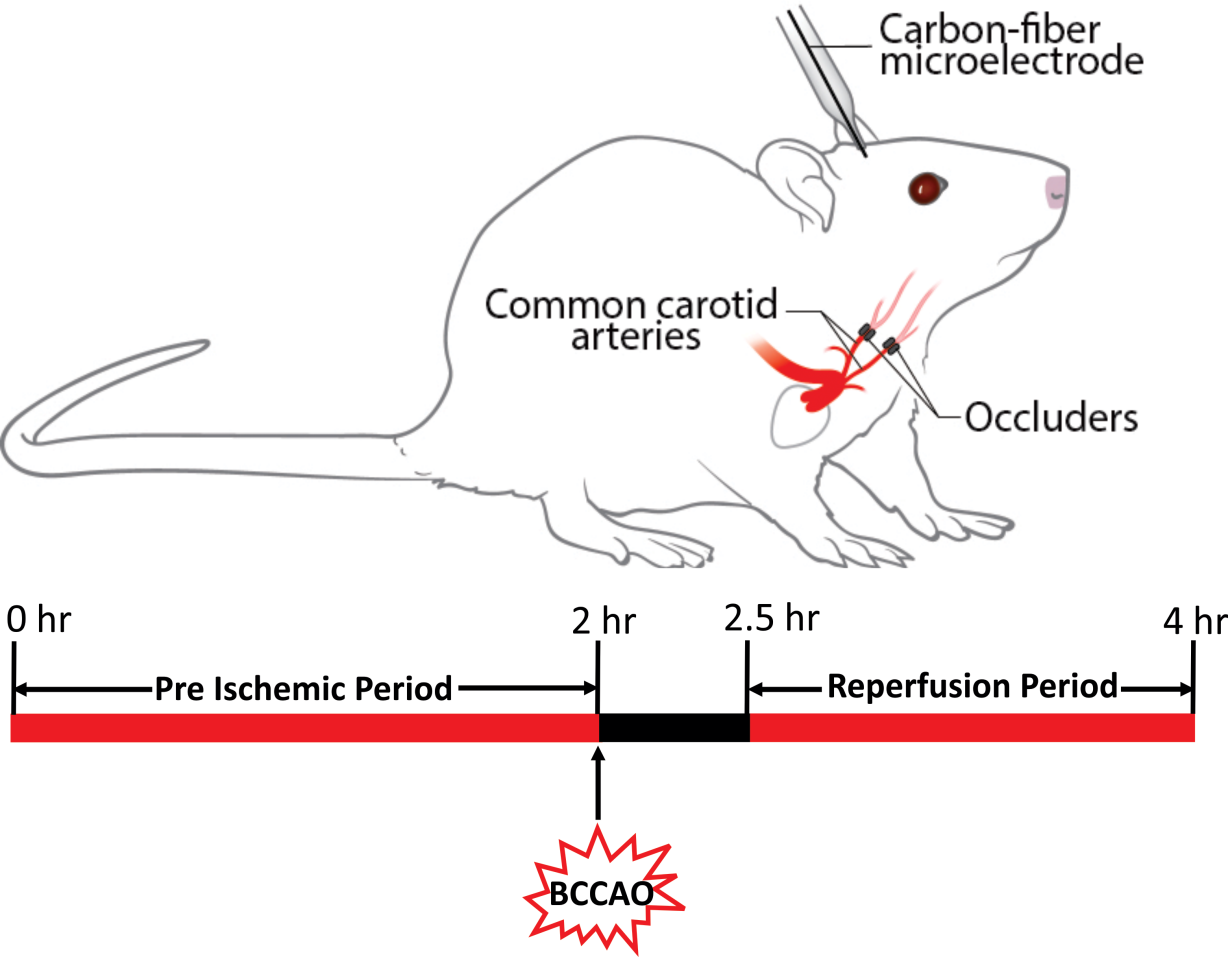
Rat schematic diagram and experimental timeline of normoxia, ischemia and reperfusion. Top: Rat schematic diagram showing the placement of the occluders around the common carotid artery and placement of carbon fiber microelectrode into the rat brain. Bottom: Timeline diagram of normoxia, ischemia and reperfusion periods.

**Fig 4:**
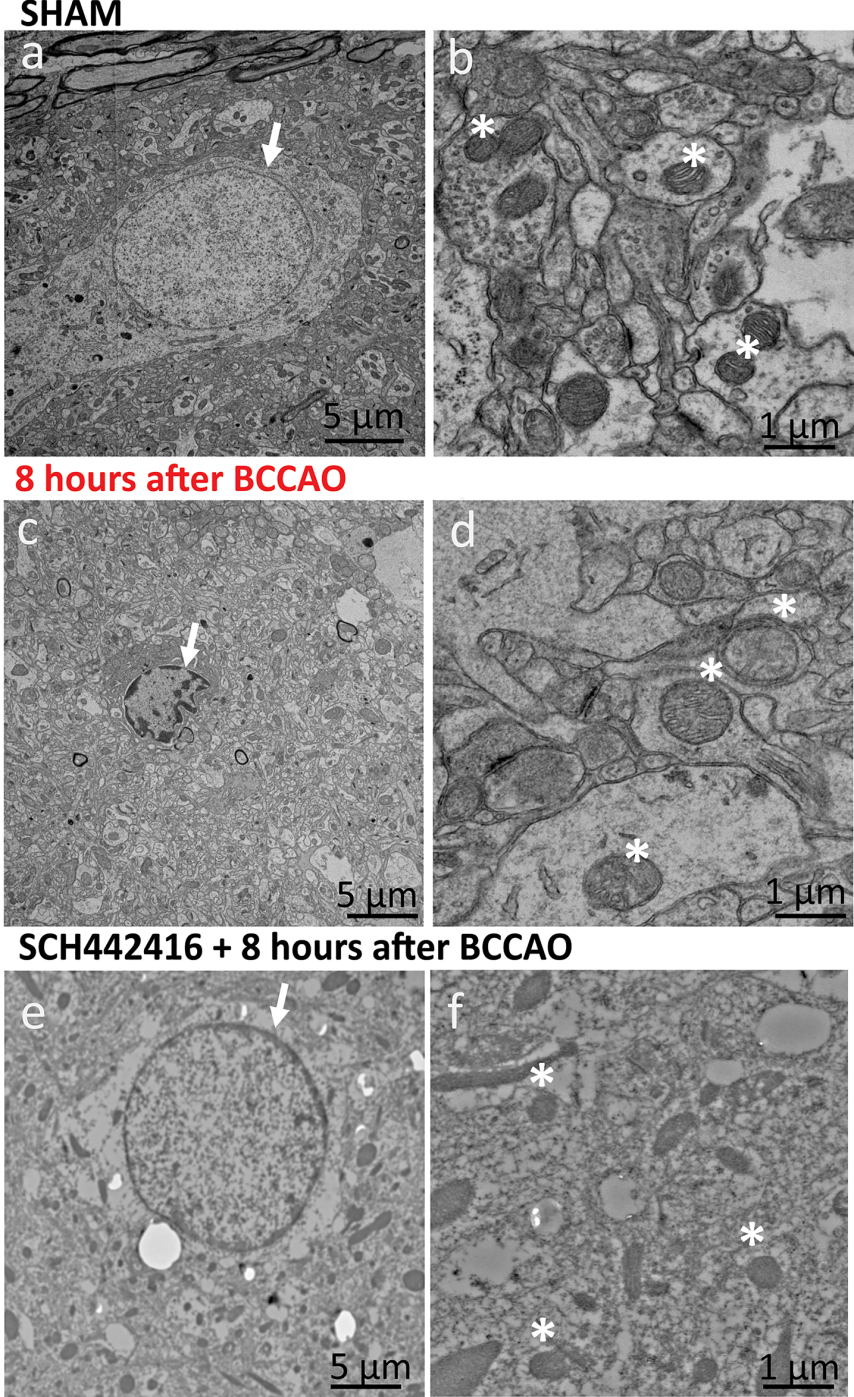
TEM images of control rat, ischemia induced and A_2A_ antagonist administered brain slices. TEM images of the nucleus from rat brain caudate-putamen. Cerebral neurons in sham rats showed (a) normal cell nucleus (arrow) and (b) normal mitochondria (*). Rats after 30 min of BCCAO and 8h of reperfusion led to substantial changes to organelle structure. After BCCAO, (c) cell nucleus appears to have shrunken with condensed chromatin (arrow). (d) Mitochondria are swollen (*) and with disorganized cristae. Rats after the administration A_2A_ antagonist SCH 442416 prior to 30 min of BCCAO and 8h of reperfusion showed (e) normal cell nucleus (arrow) and (f) normal mitochondria (*) with no disruption to cell membrane. This indicates that A_2A_ antagonist administration prior to the cerebral ischemia proven to be neuroprotective.

Bilateral occlusion of the common carotid arteries for a brief period of 30 min was used as a model of cerebral ischemia. BCCAO is a relatively mild stroke, and previous experiments have observed behavioral changes 24 hours after reperfusion, along with swelling of neuronal cells, disruption of cell membrane, and shrinkage of nucleus [31]. In the present study, cerebral ischemia was induced using inflatable vascular occluders, which are widely used in other ischemic models of rats and piglets [36]. The rat schematic diagram with the placement of occluders around common carotid arteries bilaterally and experimental timeline for normoxia, ischemia and reperfusion are shown in Fig 3. To further understand the pathological changes in neuronal cells after 30 min BCCAO, we examined brain samples using transmission electron microscopy (TEM) in sham, I-R induced rats 8 h after reperfusion, a time sufficient for damage to occur and A_2A_ antagonist administered prior to cerebral ischemia and 8 hours after reperfusion. TEM data showed that the nuclei were significantly smaller and had peripheral condensation of chromatin after 8 h of cerebral ischemia compared to those in the sham group (Fig 4). In I-R induced rats, mitochondrial structure was also affected as the mitochondria were swollen, with disarrayed cristae. Where as rats treated with A_2A_ antagonist SCH 442416 and I-R induced did not showed any abnormality in the cell nucleus or the mitochondria structure.

**Fig 5:**
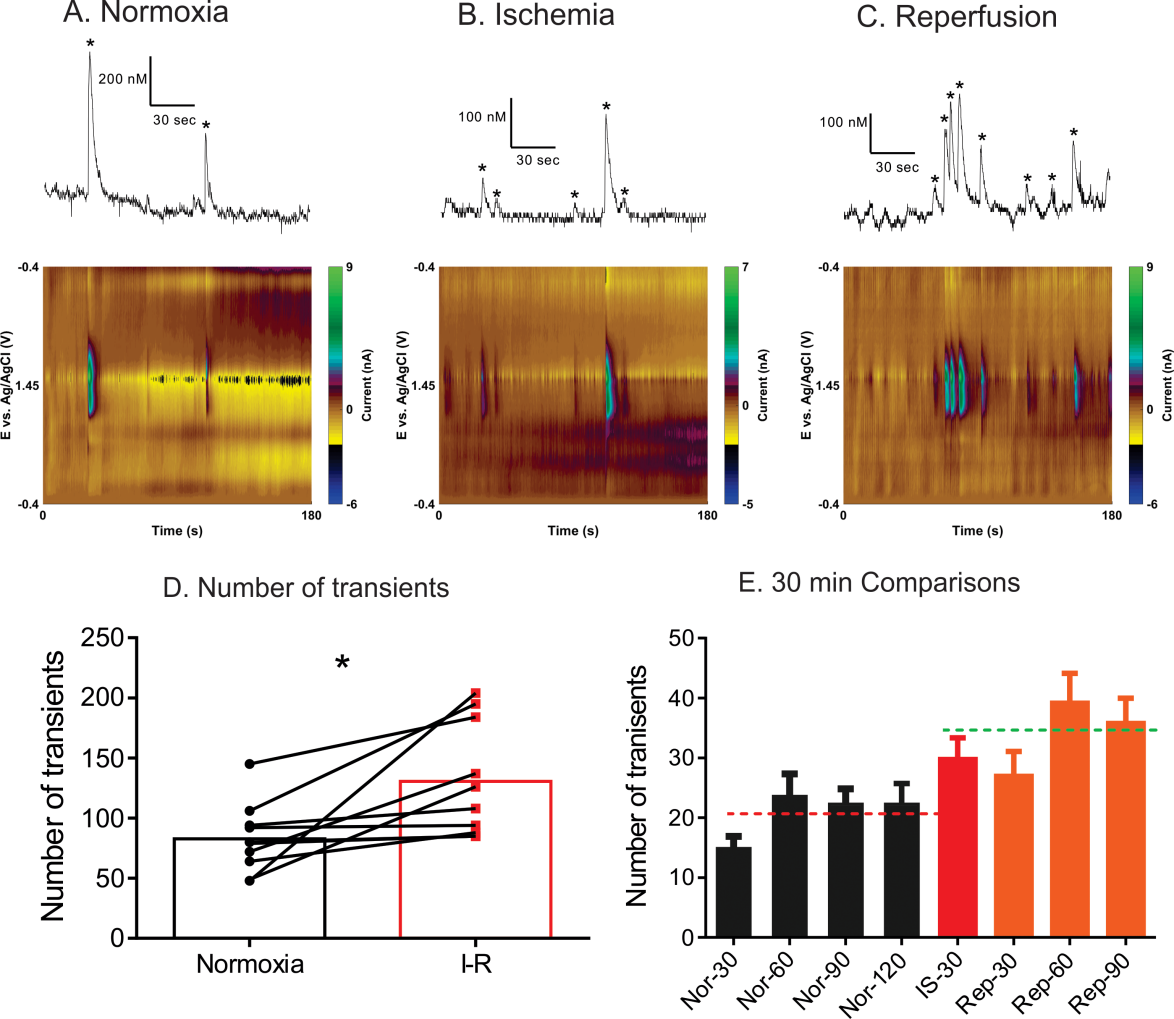
Effect of stroke on the number of adenosine transients in the caudate-putamen. All data and statistics are for n = 10 animals. Example concentration traces and false color plots showing the number of transients during (A) normoxia (B) ischemia and (C) reperfusion over 180 s time window. There are more transients during ischemia and reperfusion than during normoxia. (D) The average number of adenosine transients during normoxia (2 h) and I-R periods (2 h) is significantly different (paired t-test, n = 10 animals, p = 0.0138). (E) Average number of adenosine transients, divided into 30 min periods. The first four bars are 30 min time periods during normoxia, then one bar for the 30 min of ischemia, followed by three 30 min bars for reperfusion. The dashed lines show the average for the normoxia and ischemia-reperfusion periods. The number of transients during normoxia, ischemia and reperfusion were significantly different (One-way ANOVA, p < 0.0001).

To examine the temporal release of spontaneous adenosine during cerebral ischemia-reperfusion, spontaneous adenosine release was measured during normoxia, 30 min of ischemia, and then 90 min of reperfusion. The normoxia period is a within animal control, since adenosine transient frequency can vary widely between animals. Fig 5 shows the example of concentration trace and color plots for normoxia (A), ischemia (B), and reperfusion (C). Adenosine transients occur more frequently during ischemia & reperfusion compared to normoxia. For example, in the normoxia plot, there are 2 transients in 3 minutes, while there are 5 in the ischemia plot and 8 in the reperfusion color plot shown (all from same animal).

The total number of transients for each rat was calculated for normoxia, ischemia and reperfusion periods. The average number of transients is plotted in Fig 5D for 2 hours of normoxia and 2 hours of combined ischemia and reperfusion (I-R). The average number of transients increased significantly during I-R, from 83 to 130 (paired t-test, n = 10, p = 0.01). To further examine how adenosine was released during ischemia and reperfusion individually, the average number of transients was calculated for every 30 min period. Thirty minute bins were chosen because that is the length of cerebral ischemia, so we could separate the effects of ischemia and reperfusion. Fig 5E shows the average number of transients for four 30 min bins of normoxia, one 30 min bin during ischemia, and three 30 min bins of reperfusion. There was an overall main effect (one-way ANOVA, p<0.0001, n = 10 rats), indicating that the number of transients changes with ischemia and reperfusion periods. During normoxia, there are slightly fewer transients observed during the initial 30 min period of normoxia, but the number of transients is then very consistent. During cerebral ischemia induction, the number of transients increased and continues to be elevated during the 90 min of reperfusion. The largest number of transients was 60–90 min after reperfusion. While the overall numbers of transients are low in 30 min bins, making it more difficult to pull out statistical differences, Bonferonni post-tests indicated that the 30 min of ischemia, reperfusion 60 minute, and reperfusion 90 minute bins are all statistically higher than the normoxia 30 minute bin (p<0.05, n = 10, Fig 5E).

**Fig 6:**
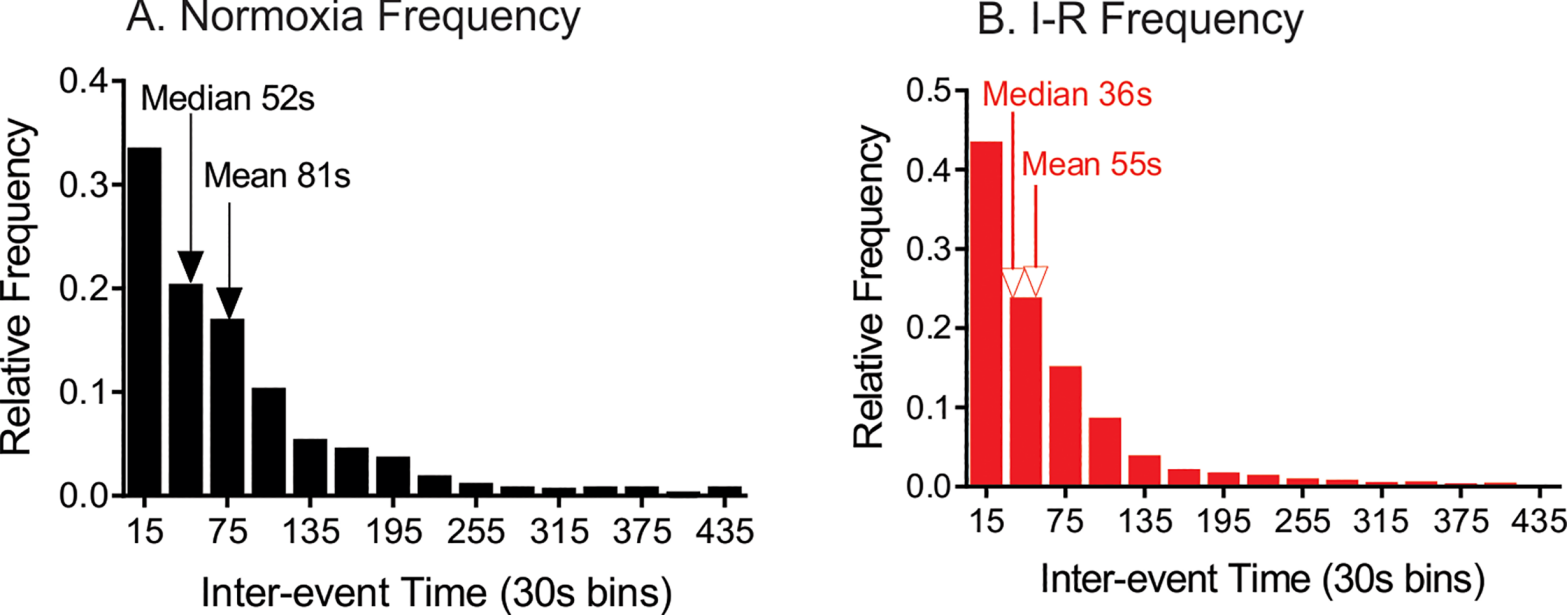
(A) Inter-event time histograms of all adenosine transients during normoxia. Median and mean inter-event times are plotted on the frequency distribution. (B) Inter-event time histograms of all adenosine transients during ischemia & reperfusion. Median and mean inter-event times are plotted on the frequency distribution. The inter-event time was significantly shorter after the induction of ischemia and there was a significant difference between the distributions before and after stroke (KS-test, n = 10 animals, p < 0.0001).

Inter-event times, or the time between consecutive events, were examined to look at the frequency of adenosine transients. On average, adenosine transients occurred frequently, about once every 50 seconds, during normoxia. Histograms of inter-event times are plotted for 2 hours of normoxia (Fig 6A) and the combined 2 hour ischemia & reperfusion (Fig 6B). There was a decrease in the median inter-event time from 52 s for normoxia to 36 s during ischemia-reperfusion, and there was a significant difference in the underlying frequency distribution (n = 10 rats, KS test comparing underlying distributions, p<0.0001).

**Fig 7:**
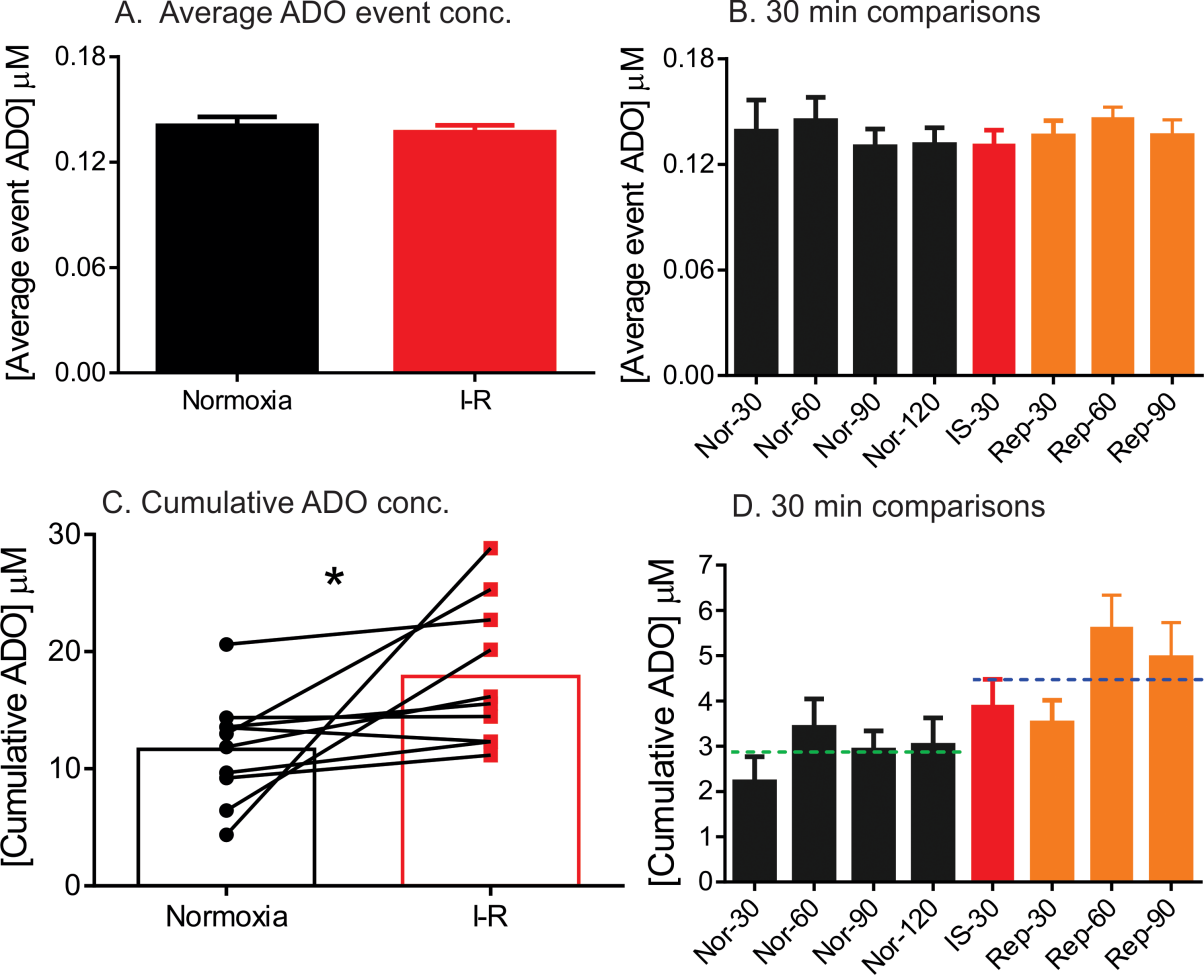
Spontaneous adenosine release frequency and concentration in the caudate-putamen during stroke. All data and statistics are for n = 10 animals. (A) The average event adenosine concentration per transient for normoxia (n = 829) and ischemia-reperfusion (n = 1306) was not significantly different (unpaired t-test, n = 10 animals, p = 0.57) (B) Average event adenosine concentration per transient were divided into 30 min periods during normoxia, ischemia and reperfusion periods and event concentration did not show any significant difference (One-way ANOVA, p = 0.9354) (C) The cumulative adenosine concentration was significantly different after stroke compared to normoxia (paired t-test, n = 10 animals, p = 0.03). (D) Cumulative adenosine concentration was divided into 30 min periods. The first four bars are 30 min periods during normoxia, then one bar for the 30 min of ischemia, followed by three 30 min bars for the total 90 min of reperfusion. The dashed lines show the average for the normoxia and ischemia-reperfusion periods. Cumulative concentration during normoxia, ischemia and reperfusion was significantly different (One-way ANOVA, p = 0.0022).

The average concentration of each adenosine event was also examined. There is a wide range of concentrations of adenosine events from 0.04 to 1.95 μM. However, the average adenosine concentration per transient exhibited no significant changes; for normoxia it was 0.14 ± 0.01 μM and for I-R it was 0.13 ± 0.01 μM (unpaired t-test, p = 0.57, n = 10 rats) (Fig 7A). To further examine whether the average event adenosine concentration changes during ischemia and reperfusion individually, the average event concentration was examined in 30 min bins. Fig 7B shows that there was no overall change in event concentration in the 30 minute bins (one-way ANOVA, p = 0.93, n = 10).

The cumulative adenosine concentration was calculated by adding up the concentrations of all the adenosine events during normoxia or I-R. The average cumulative adenosine concentration significantly increased during I-R periods compared to normoxia, with mean concentration increasing from 11.7 ± 1.4 μM to 17.9 ± 1.9 μM (paired t-test, p = 0.0373, n = 10) (Fig 7C). The cumulative concentration increase was driven by the increased number of transients during I-R and not by the change in concentration per event. To further examine the increase in cumulative adenosine concentration during normoxia, ischemia and reperfusion periods individually, the cumulative concentrations were examined in 30 min bins. Fig 7D shows the cumulative concentrations for four 30 min bins of normoxia, one 30 min bin during the induction of ischemia, and three 30 min bins of reperfusion. There was a main effect in this graph (one-way ANOVA, p = 0.0022) and the reperfusion 60 and 90 min bins were significantly greater than the normoxia 30 bin (Bonferroni post-test, p<005).

### A_2A_ receptor modulation during cerebral ischemia-reperfusion

**Fig 8:**
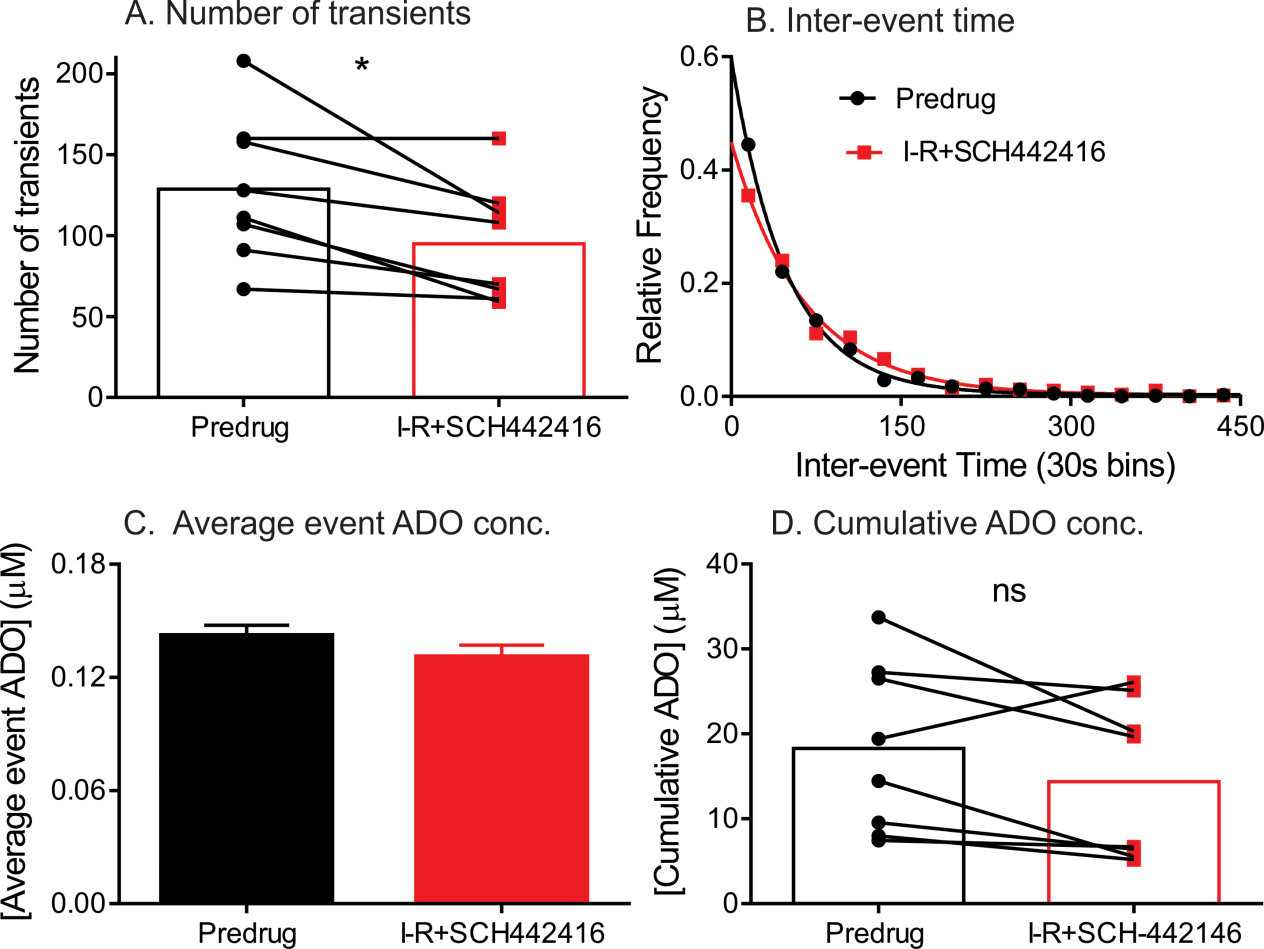
Effect of the A_2A_ antagonist, SCH442416 (3 mg/kg. i.p), on adenosine on adenosine transients during stroke. All data and statistics are for n = 8 animals. (A) Number of adenosine transients decreased significantly after SCH442416 during stroke (paired t-test, p = 0.01). (B) Inter-event time of all adenosine transients. The exponential fit (black line) during normoxia (predrug) is y = 0.5981e^-0.0207x^ (R^2^ = 0.99) and SCH442416 + ischemia-reperfusion (red line) is y = 0.4491e^-0.0155x^ (R^2^ = 0.99). After SCH442416 treatment, the inter-event time was significantly longer during stroke, with a median inter-event time change from 39 s (normoxia) to 48 s (I-R). There was a significant difference between the distributions before and after stroke (KS-test, p = p < 0.0001). (C) The average event adenosine concentration per transient after SCH442416 administration during ischemia and reperfusion 0.13 ± 0.01 μM (n = 759 transients) compared to normoxia 0.14 ± 0.01 μM (n = 1030 transients) was not significantly different (unpaired t-test, n = 8 animals, p = 0.05) (D) There was no significant change in the median cumulative concentration after SCH442416 during stroke compared to normoxia (p = 0.11).

A_2A_ receptors are highly expressed in striatum and play a neuroprotective role during hypoxia/ischemia [37–39]. A_2A_ receptor antagonists have been proposed as neuroprotective treatments and A_2A_ deficiency attenuates brain injury during ischemia [39,40], therefore, we tested the effect of A_2A_ receptor antagonist SCH442416 on spontaneous adenosine release during I-R injury. SCH 442416 was administered at 3 mg/kg, i.p, a dose that had previously showed increased locomotor activity [41]. Fig 8A shows that SCH 442416 significantly decreases the average total number of transients compared to predrug from 129 to 95 (paired t-test, p = 0.0151, n = 8). Also there was an increase in median inter-event time from 33 s to 48 s and a significant difference in the underlying distribution of transient adenosine frequency (KS test, p<0.0001, n = 8 rats) (Fig 8B). The average event concentration per transient showed no change from normoxia to SCH/I-R (0.14 ± 0.01 μM to 0.13 ± 0.01 μM, n = 8, unpaired t-test, p = 0.0568) (Fig 8C). The cumulative concentration during SCH/ I-R (13.2±3.3 μM) was also not significantly different than predrug, with concentration (16.9 ± 3.5 μM) (n = 8, paired t-test, p = 0.1069) (Fig 8D). The A_2A_ data demonstrates that by blocking excitatory A_2A_ receptors in the caudate during the course of ischemic insult attenuates the frequency of spontaneous, transient adenosine release as well as the total adenosine concentration during the course of injury.

## Discussion

This study demonstrates that the frequency of spontaneous, transient adenosine release events significantly increases during cerebral ischemia compared to normoxia and continues to be elevated through 90 min of reperfusion. Adenosine has been implicated as a neuroprotective molecule during stroke, and here we are reporting the first rapid measurements of adenosine during any ischemic insult. While the frequency of adenosine transients increased, there was no change in the mean concentration of each transient, indicating that frequency controls the neuroprotective adenosine response and not concentration per event. The higher frequency does lead to an increase in the cumulative adenosine concentration during I-R periods. While the 30 minute bilateral common carotid artery occlusion protocol used is relatively mild, not causing cell death, these data indicate that transient adenosine release is elevated during mild ischemic stress and may be an important stress signal that causes neuromodulation during cerebral ischemia and reperfusion.

### The frequency of transient adenosine release increases during cerebral ischemia-reperfusion injury

In our BCCAO model, both common carotid arteries were reversibly blocked using inflatable occluders for 30 min. The function of the includers was visually tested during each surgery by inflating for a few seconds, so we know the blood flow was blocked. Inflatable vascular occluders are advantageous compared to vascular clips, as cerebral ischemia and reperfusion can be induced, simply by inflation and deflation, while the animal is in the stereotaxic frame and adenosine measurements are being collected [36,42,43]. Previous reports have shown that induction of cerebral ischemia through 30 min of BCCAO causes significant neurological deficit and reduction in sensorimotor coordination, swelling of neuronal cells, oxidative stress disruption of cell membrane, and shrinkage of the nucleus [31,44]. We found reperfusion for 8 h led to serious edema with substantial shrinkage of the cell nuclei and swelling of mitochondria. Although carotid arteries are a major source of blood flow to the caudate-putamen, there is also collateral blood flow in the rat [45]. While this is a mild ischemic event, BCCAO still caused over a 50% increase in the number of transient adenosine release events. BCCAO is also an established model for ischemic preconditioning, which induces protection against subsequent ischemic insults [46], and this work indicates that rapid adenosine could play an important part in that induction of neuroprotection. Future work could study the real-time changes of adenosine in the brain during severe ischemia models such as middle cerebral artery occlusion (MCAO), that would cause more damage to the brain and therefore might cause more adenosine release, [47]. The majority of field is focused on MCAO models, so it would be interesting to study the temporal changes in adenosine release with more severe cell death. Our results demonstrate that changing of the frequency of adenosine transients is the main mechanism by which transient adenosine is regulated during cerebral ischemia-reperfusion. Previous studies, including those with biosensors that have time resolution in the seconds range, had not identified these rapid changes in adenosine release in in vivo models [17,22]. Here we studied the regulation of adenosine frequency during cerebral ischemia for the first time. The number of adenosine transients significantly increased during cerebral ischemia-reperfusion by 57%. Looking at the data in 30 minute bins confirms that the number of transients increased during the 30 minutes of cerebral ischemia and continued to be elevated during the 90 min. of reperfusion. The largest number of transients occurred 30–90 min after reperfusion, the critical time period where reperfusion injury might occur [48]. The variance in the number of transients in individual rats per 30 min bin is high, so there is no significant difference of reperfusion from cerebral ischemia Thus, rapid adenosine release occurs during periods where the brain is especially vulnerable to excitotoxic injury and could serve as neuromodulatory signal [49].

The average concentration of each transient adenosine event was 140 nM, although the concentrations of adenosine events varied widely from 40 nM to 1.95 μM, similar to our previous results during normoxia [23,50]. While the concentration of individual transients did not increase during I/R, the average cumulative adenosine concentration during I/R increased by 53%, because of the higher frequency of events. Indeed, the cumulative concentration during I-R, as examined in the 30 min bins, shows that micromolar levels of adenosine were release over 30 min periods after BCCAO. Thus, a key finding is that the amount of adenosine per event did not change during I-R, but the increased frequency leads to increased cumulative adenosine concentrations.

While our FSCV method measures fast changes in adenosine and not basal changes, it is interesting to compare our results with those that have reported changes in the basal levels. Early results indicated that basal adenosine levels rise within 5 seconds after an ischemic insult [51], and we also observed adenosine transients occurring right after induction of cerebral ischemia. The magnitude of the increase in basal levels varies by the type of ischemic induction and the time for ischemia. For example, middle cerebral arty occlusion produced a 5 fold increase in extracellular striatal adenosine levels to 5.6 μM during a 4 hour injury when the assays were performed 24 hours after microdialysis probe implantation [52]. With 5 min of ischemic induction using BCCAO in the gerbil hippocampus, there was no change in adenosine concentration during actual cerebral ischemia but levels rose to 13 μM during the first 20 min of reperfusion [53]. Levels measured by microdialysis may be high because these experiments were performed only a few hours after the probe implantationn and not 24 hours later, when tissue could recover. Similarly, large, 20 μM changes in adenosine concentration were measured *in vitro* during 10 minute oxygen/glucose deprivation of a hippocampal slice [7]. The new information from our studies is that cumulative adenosine concentrations of over 15 μM were observed during two hours of I-R and the frequency of transient release is what drives the increase in cumulative concentration.

### A_2A_ antagonist decreases the frequency of transient adenosine release

Because extracellular adenosine concentrations increase dramatically during ischemia, numerous studies have proposed adenosine receptors as therapeutic target for stroke [54]. A_2A_ antagonists have been proposed as treatments because they dampen the excitatory effects of adenosine, decreasing excitotoxic glutamate release [55–57]. A_2A_ receptors are highly expressed in striatum and are mostly present on GABA-enkephalin neurons [37]. They are also located presynaptically [58–60] on glutamatergic terminals, where they can directly regulate glutamate outflow under normoxia [60,61] and ischemic conditions [62].

In our study, administration of A_2A_ antagonist SCH 442416 significantly decreased the frequency of spontaneous adenosine release during cerebral ischemia and reperfusion. While the frequency of spontaneous adenosine transients increased 57% during I-R in ischemic rats, with the administration of SCH 442416, the frequency of spontaneous adenosine actually decreased 27%. The cumulative adenosine concentration also decreased after SCH 442416, driven by the lower number of transients. This is in agreement with the previous microdialysis observations that the A_2A_ antagonist SCH58261 reduces adenosine release in the MCAO model [57]. This decrease in number of transients with an A_2A_ antagonist is similar to non-ischemic rats that had a significant decrease in both the number of transients and cumulative concentration after SCH 442416 [25]. An interesting finding here is that the high dose (3 mg/kg) of A_2A_ antagonist reduced the amount of adenosine available to cause neuromodulation or neuroprotection during cerebral ischemia/reperfusion. We used a high dose to block all the A_2A_ receptors, however, previous studies have demonstrated that lower doses (~0.01 mg/kg) of A_2A_ antagonists had a protective effect in several ischemia models, and high doses of A_2A_ antagonist were less selective for A_2A_ receptors. For example, subchronic administration of A_2A_ receptor antagonist up to 15 h after focal ischemia showed significant protection against neurological deficit and infarct size [63], Our TEM data indicate that this dose did have a neuroprotective effect as cells damage induced by I/R was not as evident when 3 mg/kg SCH 442416 was administered. A_2A_ antagonists are neuroprotective because they reduce glutamate release and its excitotoxic effects. Future studies should focus on also investigating the effect of low doses of A_2A_ antagonist in this I-R model, since low doses have a proven neuroprotective effect. Adenosine also acts at A_1_ receptors to cause neuroprotection [4], so future studies that examine which receptor mediate the effects of transient adenosine release would be useful for understanding its potential neuromodulatory properties.

### Role of transient adenosine during stroke

Adenosine is well known as a “retaliatory metabolite”, linking local metabolism and cellular firing to blood flow and increased energy delivery [64]. Adenosine dramatically increases during ischemia [17], but here we demonstrate a new mechanism of increasing adenosine signaling during cerebral ischemia: increased frequency of transient adenosine events. These transient adenosine events regulate transient dopamine release and changes in oxygen, a correlate of blood flow [24,25]. Thus, this rapid mode of adenosine release may serve as a local neuromodulatory signal during stress, transiently regulating neurotransmission and blood flow both during cerebral ischemia and reperfusion. One advantage of a transient mode of adenosine signaling is that it would not saturate adenosine receptors for a long time. Thus receptors may not desensitize as quickly or may be able to continue to provide rapid neuromodulation if activated by these short bursts of adenosine.

Future studies need to examine the mechanism of adenosine formation during stroke, to determine if it is a breakdown product of ATP. Protective effects of transient adenosine during ischemic preconditioning could also be examined, as our current model of BCCAO is often used for preconditioning experiments [46]. So our current finding that even this mild ischemic stress causes an increase in the frequency of transient adenosine release that continues into reperfusion suggest that, transient adenosine may play an important role in ischemic preconditioning. While there is still much to learn about the formation and function of these transient adenosine release events during ischemia, their presence during even mild cerebral ischemia and reperfusion shows that they could be an important neuromodulatory signal in the brain during stress.

## Conclusions

We examined the early changes in spontaneous, transient adenosine release during the progression of cerebral ischemia and reperfusion injury for the first time *in vivo*. Compared to normoxia, the total number of adenosine transients and the cumulative adenosine concentration increased during cerebral ischemia and reperfusion periods. These findings indicate that rapid changes in adenosine occur during early stages of cerebral ischemia and continue during early reperfusion. Future studies investigating the protective effects of a rapid mode of adenosine signaling during ischemic preconditioning would reveal the neuroprotective role of transient adenosine during ischemic injury.

## Abbreviations

FSCV: fast-scan cyclic voltammetry
BCCAO: bilateral common carotid artery
I-R: Cerebral ischemia and reperfusion
TEM: Transmission Electron Microscopy
ADO: Adenosine
